# Student nurses as a future general practice nursing workforce. Implementing collaborative learning in practice: implications for placement learning and patient access. A mixed methods study

**DOI:** 10.1186/s12912-023-01501-8

**Published:** 2023-09-21

**Authors:** Graham R Williamson, Adele Kane, Sharon Evans, Lisa Attrill, Fiona Cook, Katy Nash

**Affiliations:** 1 School of Nursing and Midwifery, Exeter Centre, University of Plymouth, Topsham Rd, Exeter, EX2 6HA UK; 2 School of Nursing and Midwifery, University of Plymouth Cornwall Campus, TR1 3HD, Truro, UK; 3 Strategic Lead for General Practice Nursing, Cornwall & Isles of Scilly Integrated Care Board, Plymouth, UK; 4 Practice Nurse and Nurse Prescriber, Okehampton Medical Centre, Devon, UK

**Keywords:** General practice nursing, Student nurses, Placement learning, Mixed methods research.

## Abstract

**Background:**

There is a global shortage of nurses, with particularly acute shortfall in General Practice Nursing in the United Kingdom estimated at as high as 50% vacancy rate by 2031 by some sources. There has previously been reluctance for General Practices to host student nurses on placement, but it has become imperative to increase placement capacity if practices are to be able to recruit a future workforce. Collaborative Learning in Practice is a means of organising placement learning for student nurses using a coaching model, that allows for leadership development, peer support and earlier engagement in patient care, and increases placement capacity.

**Methods:**

This was a mixed methods study using qualitative data from focus groups to evaluate the implementation of Collaborative Learning in Practice, and routinely collected audit data on numbers of clinic appointments to investigate the potential impact an increased capacity of student nurses might have on patient access to services. The aims of this study were: to implement and evaluate Collaborative Learning in Practice in General Practice Nursing settings; to explore issues of interprofessional learning; to explore patient access to services related to increased student nurse capacity.

**Results:**

Our qualitative data indicated the following themes as important to students and staff: Peer Support; Interprofessional Learning; and the Importance of ‘own clinics’ for students to see patients. The audit data indicated that having students leading their own clinics increased the clinic numbers available by approximately 20% compared to when students were not in placement.

**Conclusions:**

This study shows that student nurses increased clinic capacity and improved access for patients. Students valued their placement, felt that they were more ‘part of the team’ than in other placements and consequently had a greater sense of belonging. This was multifaceted, coming in part from the welcoming practice staff, in part from the opportunities for peer support engendered by the collaborative learning in practice model, and in part from the interprofessional learning opportunities available. General Practice Nursing placements for students are important for future workforce recruitment and can help meet Quality and Outcomes Framework targets for General Practices.

## Background

Collaborative Learning in Practice (CLIP) is a method of organising practice learning for student nurses that has become popular in the United Kingdom (UK) in recent years [1]. It originated in Amsterdam with initial UK development by the team at University of East Anglia [2, 3] and support and facilitation from Health Education England [4] (HEE). HEE exists to provide leadership in education and training for the healthcare workforce in England. CLIP combines several previously novel features including students taking responsibility for patient care at an early stage of their programmes, under supervision of registered nurses (RNs, who in the UK are at least educated to Bachelor’s degree level). CLIP uses a coaching model as opposed to a mentoring model, in which the coach takes a more facilitative, structured and questioning approach to student supervision and assessment when compared to the more individualised approach of the mentor [3]. Students receive training prior to starting placement about how the CLIP model works. Once in placement, students typically work in small teams, often including third year students taking a leadership role at the head of a group of students including first, second and third years, as well as Trainee Nursing Associates who undertake a two year foundation degree programme leading to NMC registration, and unregistered nursing staff such as Health Care Assistants (HCAs) [5]. CLIP was initially implemented in hospital settings, where wards were required to increase placement capacity [2], and was found to have a positive impact on patient safety [6]. Latterly, CLIP has also been trialled in community settings [7], children’s mental health care [8], maternity care [9] and physiotherapy placements [10] in the UK. There seems to be no international equivalent term to CLIP, but the concept of Dedicated Education Unit (DEU) appears to offer similar potential for collaborative Interprofessional Learning (IPL) facilitated by clinical coaches [11]. Internationally, projects report success with a collaborative approach to IPL for community care in Singapore [12] and with dental students in Canada [13], and it is also clear that general practice is a fertile ground for IPL [14, 15].

A common international thread through healthcare and nursing literature is that of staff shortages [16] [17]. As well as global shortages, the World Health Organisation’s (WHO) State of the World’s Nursing report [18] also calls for practitioners capable of working in community settings at the point of registration. In the UK, it is estimated that there was a shortage of 46,828 nurses in June 2022, meaning that in some regions approximately 20% of nursing posts are unfilled. Failure to invest in General Practice Nursing (GPN) recruitment and training is projected to see UK-wide shortfall of around 6400 nurses, more than one in four posts, by 2030-31. More pessimistic modelling indicates this could be as high as one in two vacancies unfilled in that time frame [19].

A central drive in the popularity of CLIP is that it increases placement capacity, with clinical areas in our previous studies supporting approximately three or four times the numbers of students in CLIP placements than at other times [2, 6]. Elsewhere it is noted that CLIP functions using a coaching approach to placement learning, as distinct from the mentoring approach in evidence when there was only one student alone in placement [3]. It is argued from qualitative findings, that introducing student nurses to direct responsibility for patient care and leadership from their third year ought to lead to better preparedness for registrant practice [1, 3], but this has yet to be more formally evaluated with new graduates.

Access to GPN placement experience for students has traditionally been problematic when practices have not seen a direct benefit or financial incentive to host them [20]. The National Health Service England (NHSE) Sonnet Report on the strategic value of GPNs asserts that education and training are vital to the future GPN workforce, and that student nurses bring particular benefits to practices [21]. It is axiomatic that student nurses are the future workforce of any organisation, and that recruitment needs to be strengthened [22]. This is represented in the NHSE GPN 10 point plan, designed to increase recruitment and retention of GPNs [23] and in our region much work has been done with GPs, GPNs and practice managers to enable students’ access to placements within local Primary Care Networks (PCNs). When this study began, PCNs were groups of practices designed to develop and deliver existing primary care services to patients, involving proactive and coordinated multidisciplinary care, and synergies commensurate with economies of scale [24]. In the UK, GPs are in a unique position as part of the NHS, existing simultaneously as independent contractors. GPs are incentivised to deliver services benchmarked to a national standard, through a national GP contract [25], which is assured through the Quality and Outcomes Framework [26], and this includes detailed standards relating to patient access and standards of expected care.

Research has indicated that student nurses do not necessarily see GPN as an appropriate job destination on graduation, but also that exposure to GPN can have a positive influence on their perceptions of it as a first destination [27, 28, 29]. As a result, Health Education England (HEE) has developed a Workforce Plan [30], which specifically calls for a greater visibility for GPN in nurse education, increases in the numbers of student nurses accessing GPN placements, and proposing GPN roles as a first destination, as well as appropriate education pathways for careers after registration.

Having outlined the international, national and local context of shortages of nurses in GPN settings, pressures to recruit nurses and make GPN an attractive first job destination, we argue that it is appropriate to implement and evaluate CLIP in GPN placements in our regions, and this paper reports a project undertaken to address those issues.

## Methods

### Aim

The aims of this study were threefold.

1. To implement and develop CLIP in GPN settings and evaluate that implementation.
2. To explore issues of interprofessional learning in GPN settings.
3. To explore issues of patient access to services relating to CLIP and increased student nurse capacity in GP practices.

### Design and setting of the study

We placed 31 student nurses into six GP practices in three PCNs; this being 15 students in Winter 2021 and 16 students in Summer 2022. Previously (pre-CLIP) one or two of those practices might have had one or two students in total. This was a mixed methods study using qualitative data from focus groups to evaluate the implementation of CLIP, and routinely collected audit data (meaning anonymous monitoring data that would have been collected out-with this research project) on numbers of clinic appointments to investigate the potential impact that an increased capacity of student nurses might have on patient access to services. We used a mixed methods approach because it was an appropriate study design to access the beliefs of students and staff across several locations and times in our qualitative Microsoft Teams focus groups (FGs), coupled with quantitative data about patient access to GPNs. This study took place in GP surgeries in three counties in the Southwest of England. Students and staff all received training about CLIP and their roles, including supervisory training for staff. One important characteristic regarding these students’ placements is that students received training in venepuncture immediately prior to their placements, meaning that they could take blood from patients in phlebotomy clinics. After a period of familiarisation, students were also able to lead their ‘own clinics’ under indirect supervision (called ‘CLIP clinics’) from an early stage in the placement, honing their skills through the ethos of coaching within CLIP placements. There was a weekly Friday meeting where students and supervisors met to debrief, reflect on their learning, and set learning goals for the following week. How this was organised varied between practices, but it typically meant that student nurses would be seeing patients independently, with their student status known and available for patients, so that they could undertake simple procedures like blood tests, hypertension checks and uncomplicated dressings that otherwise would have been undertaken by other staff, registered or unregistered. Direct or indirect supervision from Registered GPNs was always available, however these GPNs were not routinely in the same room as the students, although they could be present immediately if required. Opportunities for discussion and debriefing of students were available through the working day, as well as at the Friday meetings.

### Data collection and analysis

Data collection took place during two periods: January 2021 and July 2022. These were periods when student nurses were on placements in the GP practices.

For Aims 1 and 2, we conducted separate Microsoft Teams FGs with students and GPN staff to explore their experiences of CLIP working and interprofessional learning. Focus groups are semi-structured discussions with groups of people who explore issues of joint interest, and rely on interaction between group members to share experiences as part of offering understanding of the topics in question, as group interaction encourages respondents to explore individual and shared perspectives [31]. MS Teams on-line platform was enabled to generate transcripts. These transcripts were anonymised by omitting any identifying features such as names and locations and the recordings locked so that they are not accessible except to the lead researcher. The qualitative data analysis involved the following steps: familiarisation and construction of initial themes or concepts; indexing, labelling, and tagging the data to construct links between categories by sorting them according to levels of generality and employing a hierarchical structure so that themes and subthemes start to emerge; followed lastly by descriptive analysis, where the themes are refined, finalised and agreed between the research team [32, 33]. As a step to reduce potential bias and enhance rigour, this data analysis process was undertaken independently by two researchers, who then met to discuss and agree the final themes and subthemes based on inferential reasoning [34].

For Aim 3 regarding issues of patient access to services, we used routinely collected audit data, anonymised at source from one GP practice as a case study, to quantify the difference that having student nurses in CLIP made in terms of patient access to appropriate appointments.

### Ethical issues

The project had approval from the Faculty of Health Research Ethics Committee and permission to proceed via the UK Health Research Authority (HRA) Integrated Research Application System (IRAS). This was extended from an earlier qualitative study to include all the data collection methods used in this study. IRAS project ID: 259485. All potential participants were contacted by professional email addresses and given the Participants’ Information Sheet which included details of the study, and a consent form. Students and staff were given the usual guarantees for confidentiality, anonymity and right to withdraw, and were asked to sign written consent to take part in the MS Teams FGs. Study information and consent to participate was re-iterated prior to the FGs, and the initial minutes of the recordings document that all those who participated understood and consented to the study. No participants subsequently asked to withdraw data. This study was funded by Health Education England with an ad hoc grant.

## Results

### Characteristics of participants

The student nurses were all female except for one male. All the GP placement staff were registered nurses with experience in student support as well as in GPN. All staff were female. We conducted two FGs with student nurses, one in January 2022 and another in July 2022. Of 31 students invited, seven attended in Jan 2022 and another five different students attended in July 2022. With regards to staff data collection, we conducted two FGs with placement staff, one in January 2022 and another in July 2022. Of 42 GPNs invited, five attended in Jan 22 and a further six attended in July (one staff member attended both groups).

### Qualitative findings

Table 1 summarises the final themes and subthemes from the qualitative data analysis. Indicative quotes are included in the following analysis. These are coded so that anonymity is maintained, referring to the status of the respondent and which FG they took part in, so for example the suffix ‘Staff FG 1 Participant 2’ indicates that the quote comes from the second staff member speaking who was taking part in the first FG.

**Table 1:** themes and subthemes from the qualitative data analysis

Theme	Subthemes
Peer Support	Psychological support Helped with learning Leadership for third years
Interprofessional Learning	Breadth of experiences Multi-disciplinary team case management Sense of belonging
Importance of ‘own clinics’	Enhanced Responsibility Validity as a job destination (students) Coaching (staff)

### Theme 1: peer support

This theme was central to the discourse in staff and student focus groups in both time periods. It is clear from our findings that these students had spent most of their previous placement experiences working in environments where they were the only student or, if there were other students, they did not engage much with them. Students had felt inhibited in their professional relationships with RNs and other staff, and valued the opportunity that this GPN CLIP placement offered them to interact with other students, share their experiences and work collaboratively, whilst knowing that there was appropriate supervision available close by. The subthemes that emerged in this theme were Psychological support; Helped with learning and Leadership for third years. Regarding Psychological support, the following quotes are indicative of how students and staff perceived the benefits of CLIP with its increased capacity:

It’s nice when you’re working alongside other students, and you know that you’ve got that Friday where you’re all together in the same place and you just kind of offload to each other about the week. And it’s like a way of having a massive, deep group brief as a group, isn’t it? Yeah. Because we’re all in the same position. (Student FG 2 Participant 2).

This was echoed by staff:

They also encourage them to support each other a lot more, so they formed that sort of close relationships. So, if there was a problem on a shift or even outside of a shift… they really did communicate well between themselves. (Staff FG 1 Participant 1).

It was clear that students also valued being able to learn from each other and that being together facilitated that, in line with a second subtheme of ‘Helped with learning’ shared by staff and students across both FGs:

I felt learning from other students was brilliant. I think it boosted my confidence massively. (Student FG 1 Participant 2).

And this was reflected in the staff data:

I think that the way they do CLIP now and the way they collaborate together [is much better]. I see them learning much more than I used to…having them to bounce off each other. I think it teaches them a little bit more, to be fair. (Staff FG 2 Participant 1).

One further subtheme that emerged from the data concerned the leadership roles that third year students were able to perform with more junior students:

It was good for me as well as a third year… when I first started on the GP surgery, I didn’t really know what I was doing. And then having [junior students] coming to us and asking us questions about what we thought, that boosted my confidence, “no, actually, I do know what I’m talking about, and I’m ready to maybe be a [registered] nurse”. (Student FG 1 Participant 4).

And this was echoed in the staff data:

If a second year is struggling with something that the third will give them encouragement, you know, that’s quite a benefit…it depends on their background but they all will support each other. (Staff FG 2 Participant 2).

### Theme 2: interprofessional learning

Within the IPL theme, the first sub theme concerned the Breadth of Experiences that were on offer in the GP placement when students were able to link up and work with a wide range of professionals, and in different ways than might be possible in an in-hospital placement or another community setting. For example, students listed themselves as spending time with and learning from: GPs, Advanced Nurse Practitioners, Dieticians, Community Midwives, District Nurses, Physiotherapist, Podiatrists, Specialist Elder Care Nurses, Pharmacists, Well-being Coach, as well as un-registered healthcare staff. Students planned these liaisons themselves, and these were seen as good learning experiences by staff:

[Students] having access to the clinics and PCN staff… they can plan their own week without us having that responsibility of making sure they’ve actually got something allocated every day. If they haven’t got a CLIP clinic on, they have taken responsibility for their own allocation, their own learning objectives. (Staff FG 1 Participant 2).

And students noted how different their participation in learning was in the GP setting compared to hospital placements:

As soon as you go into hospital [placement], you can’t really do anything. So, you’re just sort of watching it. (Student FG 2 Participant 5).

In all cases this IPL was facilitated by students’ exposure to ‘Multi-Disciplinary Case Management’. The staff were clearer how this operated and what nursing involvement could be:

We have an 11:00 o’clock meeting, which, anybody [any healthcare professional or student] can come to … we can talk about patients or talk about the home visit that somebody might doing… it’s good for all sorts of things, but it’s good to hold the team together and it does help with the students. They [students] just bring another dimension to it and other you know, it’s another voice. (Staff FG 1 Participant 4).

Students detailed how they were involved in IPL as they were given project work to do, and this student shows how students collaborated and learned from the experience of investigating patient journeys, eventually meeting one patient:

The first week or second week we were given a couple of case studies to look at and then we actually met one of them [patient]… We could ask questions and different things in relation to how he felt he was supported by the surgery and other community people [healthcare professionals]. We also had another patient…and we had to sort of determine how he was diagnosed; it was over three or four years. And it’s amazing that he’s still alive today to be honest. But yeah, I met him in the GP surgery. I think the week before I left. (Student FG 1 Participant 1).

The third subtheme relating to IPL and the students’ experience of working in GPN CLIP placements was how they felt a greater ‘Sense of belonging’ in the teams in which they worked.

This student makes it clear how she felt more valued in her GP placement, and this type of dialogue was echoed by almost every student in this study:

The staff treat you a lot differently in primary care than in acute. We like the staff, but that the GP surgery and [name] are amazing. They’ve all been so lovely, including the doctors as well. And I think that makes a massive impact to your experience. (Student FG 2 Participant 1).

This quote from a staff member illustrates how the students were valued and how staff worked to make them feel welcome and that they belonged:

[The GPs] love it to be fair, one of our partner GPs, he can’t wait to call in a student [nurse] and teach them about ECGs… The GP and the other clinicians, they like sharing what they know and encouraging them. Yeah, they really enjoy it. And the reception team do as well. They like having young people around because most of us are ‘getting on a bit’ now. (Staff FG 2 Participant 3).

### Theme 3: importance of ‘own clinics’

Throughout the dialogue from staff and students, the issue of ‘how’ students worked in the GPN placements was mentioned. This was in their ‘own clinics’ (called ‘CLIP clinics’) and from that concept flows several other factors and benefits that accrued to staff and patients and impacted on the way in which staff worked with students. This had implications for students’ confidence as independent practitioners, as well as having an impact on their relationship to GPN as a potential job destination, and for improving patients’ access to certain services which students were able to deliver. In this overarching theme, staff and students in their respective FGs discussed ‘Enhanced responsibility’, but there was a distinct dichotomy between staff and students in other subthemes, such that it is justified to report staff and student data separately for the only instance in this paper. Staff discussed how students working in their ‘Own clinics’ meant they were using supervision at a distance in a coaching approach, but staff did not discuss any impact that the placement may have had on students’ job choices on qualification. Students discussed how this GPN placement has fostered a desire to work in GPN, but they did not discuss coaching as a placement learning strategy.

The first subtheme ‘Enhanced responsibilities’ relates to how students and staff perceived that GPN placements for these students had offered them enhanced opportunities to take responsibility for patient care that often were not available from in-hospital placements, largely because they were seeing patients in their ‘own clinics’, as well as because they were undertaking a range of activities with patients that are elements of the GPN role, many of which are listed below:

We found that [having our own clinics] an amazing experience…we did lots of things. And as the weeks went on, we added different stuff to our clinics, so it started out with… basic blood pressure, hypertension reviews, diabetic reviews and in the end, we were doing maybe more complex dressings assessments, ECGs, flu vaccines. We were doing a lot. We found it really good and nice to learn in our own space. (Student FG 1 Participant 2).

This was echoed in the staff perceptions:

I think for all the practice nurses as well, we were quite concerned that [students] would be running a clinic, seeing their own patients. We wouldn’t be in the room; it’s just something about having control, isn’t it? Now we find it brilliant, because we spend the first couple of weeks training them up and working with them so that we are familiar a bit more with their capabilities. (Staff FG 1 Participant 4).

It was clear from staff data that they were using a ‘coaching’ approach in which students were facilitated to be at the centre of care delivery within their competencies, and that the students being independent in their ‘own clinics’ was central to that, as this exchange shows:

If you’ve got multiple students in general practice, it has to be a coaching approach because you don’t have the capacity to be able to mentor, you can’t just have one person on a 1 to one because we don’t have the capacity for that. (Staff FG 2 Participant 2).

I agree with that. Yeah. You it is. It’s naturally a coaching thing. Our students sit in with us for the first couple of weeks before they have their own clinics. (Staff FG 2 Participant 3)

Students discussed how this placement and exposure to GPN had helped them to identify its ‘Validity as a job destination’, which was their second subtheme. This dialogue took place in focus group 1:

You know, I never considered general practice nursing. Yeah, after the after [this] placement it is an option that I would consider now, definitely. A lot of students they don’t consider that when they graduate. (Student FG 1 Participant 6)

Having reported qualitative data regarding Aims 1 and 2, we will now report results for Aim 3 relating to patient access and audit data results.

### Patient access audit data results

Regarding Aim 3: in one PCN that included two practices in one South West County, between November and December 2021, student nurses in this CLIP placement ran an additional 200 clinic appointments, so the total number of clinic appointments rose from 1060 to 1260, an increase of 20%. Students in these clinics saw patients for blood tests, long-term condition monitoring reviews, observations including hypertension reviews, simple dressings, ECGs. (Students did not review medications in their clinics). Also, 65 additional COVID vaccinations took place when student nurses were available. Anecdotal evidence from the PCN data manager indicates that having student nurses in CLIP meant that some patients were able to access same day appointments for blood tests when otherwise there could be a two week wait. This was supported in the FG data analysis.

### Data synthesis regarding patient access and skill mix

Although not well developed enough to stand as a coherent theme or subtheme of the qualitative data, staff and students did allude to issues of patient access and skill mix in the focus group discussions, in ways that support our analysis of the increased clinical appointment opportunities detailed above. As this study took place at the end of COVID-19 pandemic in the UK, when physical access to GP services had been restricted for many months (even though it was available in ways other than direct contact), this was an issue being discussed in the media in the UK. Staff discussed how students could see patients in person and spend much more time with them and outlined how student nurses taking their ‘own clinics’ could free up other professionals to do other things. Students did not go into detail about this but were clearly able to take on activities such as venepuncture for the patients attending ‘their’ clinics that might otherwise have been the preserve of HCAs or phlebotomists. The extent to which student nurses had an impact on the skill mix in the practices in which they were placed is also unclear. However, it was clear from the audit data that there was an increase in capacity of clinic appointments available.

## Discussion

This paper has detailed our mixed methods study concerning the implementation of a CLIP placement learning approach in GPN settings. Our findings indicate that the project was successful in increasing capacity, and that students valued their time in general practice, believing that it enhanced their learning by enabling peer support, interprofessional working and beneficial responsibility when practicing in their ‘own clinics’. It is also clear that these findings are despite the impact of COVID and because staff were able to facilitate students’ learning effectively. Although running their ‘own clinics’ would appear to individualise students’ practice, when opportunities were facilitated or available for sharing and group working (in Friday CLIP sessions and MDT meetings), students took these opportunities to work together and support each other. Peer support has been a consistent theme in our own research [2, 7] and that of others [3, 9] as a key benefit of CLIP. Indeed, it is noted as being central to effective clinical learning in international literature [35, 36] with particularly strong benefits for third year student nurses because they can rehearse leadership roles in the small teams of students in which they learn [35]. Going through formative professional experiences with other people at a similar stage in their development is powerful in fostering effective learning, and can contribute to individual students’ decisions about whether they stay on their programmes of study, graduate and enter the workforce [37]. In our study this is coupled with a greater sense of belonging, which was a product of the reportedly more welcoming cultures in these GP surgeries in which the students were placed. Belongingness is a driver in human motivation that influences health and well-being, and when successful, the attachment an individual student nurse experiences to a particular placement is powerful in fostering learning and interpersonal relationships. In our study, the warmth that students experienced from placement staff helped them to perceive themselves as being integral to service provision [38]. Conversely, research has indicated that placement incivility from staff mitigates against a sense of belonging in students [39], and that when students’ sense of belonging is low, that is a predictor of stress in placement [40, 41] which in turn is a factor in their decision to leave their programmes of study [42, 43]. Based on our study findings, we believe that staff in these GP practices worked hard to make students feel part of the team and engender a sense of belonging, and that this personal support from placement staff was beneficial to student’s learning and overall satisfaction.

It must be noted that our students and staff did not report significant disadvantages or negative issues related to their placement experiences, certainly nothing sufficient to constitute a theme or subtheme in our data analysis. We attribute this to the fact that placement staff, students and university personnel have considerable experience of implementing CLIP and facilitating placement learning using it, as CLIP has been used previously in our region. We have evaluated these developments in our programme of HEE-funded research and noted more critical dialogues in those research publications [2, 6, 7].

It also appears from our findings that there was a clear recognition of the potential for GPN placements to foster interprofessional learning, and that students were exposed to this on a regular basis. Communication, leadership and training are essential for successful IPL [15], which has long been identified as a feature of community practice settings in the international literature, is discussed as an essential component in multi-disciplinary working [44], and has been linked to beneficial outcomes for patients including wound healing [45], medicines optimisation [46], holistic care and identification of social determinants of health [47], and in managing multimorbidity [48]. In our study, student nurses valued opportunities to learn from a myriad of healthcare professionals and reported that they were welcome to contribute to formal and informal IPL experiences. IPL has been shown to benefit critical thinking, teamworking and cooperation amongst student nurses and other neophyte professionals [49], and so we argue that our GPN settings functioned as exemplars of how interprofessional care can be achieved because they fostered a team vision and shared goals, and sense of belonging to the team [50].

Lastly, our study uncovered evidence that increasing the capacity of student nurses across a PCN could improve patients’ access to the services that they were able to deliver (under supervision). These services included blood tests, patient monitoring reviews, simple dressings, observations and ECGs. In the one PCN from which we received data, an additional 20% clinic appointments were created, and an additional 65 COVID vaccinations took place when student nurses were available. We were not able to formally evaluate this across the region, but having student nurses made a difference to patient access, may alter the skill mix so that RNs and HCAs can do other things, and this can help meet the Quality Outcomes Framework (QOF) and the GP contract [25, 26]. For example, the QOF guidance for 2022/23 [26] contains detailed requirements to optimise access to general practice and includes activities including continuous quality improvement and network participation to improve access, which would be helped by GP practices accepting greater numbers of placements for student nurses in CLIP configurations. Specific disease conditions and patient groups are listed in the outcomes [26]: regarding obesity and weight management, the concomitant multimorbidity that obesity generates could benefit from student nurses’ involvement in taking observations, advice and referrals as part of their own ‘CLIP clinics’; and the greater number of clinic appointments available would improve access for these patients. We argue therefore that our student nurses help to improve access for patients and that this is in line with the GP contract and QOF requirements [25, 26]. This shows tangible benefits for GPs, GPN leaders and practice managers to develop placements for nursing students and to increase their capacity. Advice and guidance are available on how to make such placements effective [51]. Support from Registered GPNs as a profession for the expansion of placement provision for student nurses and consensus that GPN recruitment depends on expanding placements [21] and central direction from NHS about capacity development [23].

### Limitations

Although this study had a relatively large sample size and geographic distribution, it must be acknowledged that it took place in one UK region, with students from one University School of Nursing and Midwifery, and with GPN staff, most of whom were (broadly speaking) active in their engagement with CLIP and supportive of what the project was trying to achieve. This is a feature of much qualitative research; however, we believe that we have taken the necessary steps to ensure transparency and rigour in relation to the data collection and analysis processes we have undertaken.

In relation to the quantitative audit data, we acknowledge that this is from one PCN only. Efforts were made to obtain similar anonymous audit data from across the region, but this was unsuccessful. We therefore make no claims that the magnitude of the improved access is generalisable, however we do believe that additional students running their own CLIP clinics (under supervision) would make a difference, with patients able to access some services quicker than if the students were not there.

## Conclusions

In the context of international shortages of nurses, and of UK national shortages of GP practice nurses potentially reaching a ‘worst-case’ scenario of 50% vacancies unfilled by 2031 [19], it is imperative to attract more registered nurses to this occupational group. Students in our study discussed how their GPN experiences had made them see it as a potential job destination, partly because of the better sense of belonging that they encountered, but also because of the range of activities they were able to undertake and the relative autonomy they enjoyed. Even though we are pleased to report that three of the 31 students in this study have subsequently secured their first registered nurse jobs in GPN, we have not formally quantified an impact on first job destination in this study but, like previous studies [28, 52], our findings indicate that a placement could change student nurses’ attitudes towards community working and encourage them to apply there as a first job destination on graduation. We recommend further research in this area with students approaching graduation, and those newly qualified for less than a year, to evaluate how best this can be achieved.

Our study also shows that a strong element in the placement was IPL, and so we argue that GPN placements might be considered as an exemplar of IPL in healthcare education [12], and that this could have substantial benefits to patients in the future, although again we have not quantified this. We recommend further research with a focus on IPL and GP surgeries, and the impact that using CLIP as a model for placing all student healthcare professionals might have.

Lastly, we demonstrated that having an increased capacity of student nurses in a placement could improve access to services as students were able to lead their own clinics (under supervision). This is likely to have benefits to patients’ access, but we have not collected data from patients about their experiences with students, and we therefore recommended further research to illuminate what patients think about seeing students. ‘Access’ is a problematic area in the UK currently, and any improvements would be welcome, particularly if they reinforce care given by PCNs, support recognition via the QOF and GP contract [25, 26], and enhance the standing of general practice [23].

## Data Availability

The datasets analysed during the study are available from the corresponding author on request. Data are not public for reasons of confidentiality.

## References

[1] Harvey S, Uren CD (2019). Collaborative learning: application of the mentorship model for adult nursing students in the acute placement setting. Nurse Educ Today.

[2] Williamson GR, Kane A, Plowright H, Bunce J, Clarke D, Jamison C (2020). Thinking like a nurse’. Changing the culture of nursing students′ clinical learning: implementing collaborative learning in practice. Nurse Educ Pract.

[3] Hill R, Woodward M, Arthur A (2020). Collaborative learning in practice (CLIP): evaluation of a new approach to clinical learning. Nurse Educ Today.

[4] Health Education England. Case Study: implementing collaborative learning in practice - a New Way of learning for nursing students. In: eWIN Workforce Information. 2017.

[5] Lobo C, Paul R, Crozier K (2021). Collaborative learning in practice coaching to support student learners in healthcare.

[6] Williamson GR, Kane A, Bunce J (2020). Student nurses, increasing placement capacity and patient safety. A retrospective cohort study. Nurse Educ Pract.

[7] Williamson GR, Bunce J, Kane A, Jamison C, Clarke D (2020). Investigating the implementation of a collaborative learning in Practice Model of Nurse Education in a Community Placement Cluster: a qualitative study. Open Nurs J.

[8] Hirdle J, Keeley S, Allan L, Bareham S. A collaborative learning model for student nurses in child mental health. *Nursing Times [on-line]* 2020, 116.

[9] Markowski M, Yearley C, Bower H (2022). Collaborative learning in practice (CLiP) in a London maternity ward-a qualitative pilot study. Midwifery.

[10] Morris J, Corp G, Morris J, Robinson A, Wyndham-Birch N, Wilson D, Royle H, Modrate M (2022). Four-strands of qualitative study understanding the perspectives of students, clinicians, senior managers and leaders on collaborative-learning-in-practice (CLiP) on physiotherapy placement. Physiotherapy.

[11] Williamson GR, Plowright H, Kane A, Bunce J, Clarke D, Jamison C (2020). Collaborative learning in practice: a systematic review and narrative synthesis of the research evidence in nurse education. Nurse Educ Pract.

[12] Lau ST, Liaw SY, Lau Y, Lopez V. Seeing beyond the expected. Nursing students’ experience in community practice and collaborative learning: a qualitative study. Health Soc Care Community 2022. 10.1111/hsc.13868 35698735

[13] Kanji Z, Lin D, Karan J (2020). Assessing dental hygiene students’ readiness for interprofessional learning and collaborative practice. J Dent Educ.

[14] Toth-Pal E, Fridén C, Asenjo ST, Olsson CB (2020). Home visits as an interprofessional learning activity for students in primary healthcare. Prim Health care Res Dev.

[15] Hughes LA (2014). Qualitative evaluation of general practices developing training for a range of health disciplines. *Education for primary care: an official publication of the Association of Course Organisers, National Association of GP Tutors*. World Organisation of Family Doctors.

[16] Marć M, Bartosiewicz A, Burzyńska J, Chmiel Z, Januszewicz P (2019). A nursing shortage – a prospect of global and local policies. Int Nurs Rev.

[17] Tamata AT, Mohammadnezhad M. A systematic review study on the factors affecting shortage of nursing workforce in the hospitals. *Nursing open* 2022. 10.1002/nop2.1434 PMC9912424 36303066

[18] World Health Organisation. State of the World’s Nursing 2020: investing in education, jobs and leadership. In. Geneva: World Health Organisation; 2020.

[19] Wise J (2022). England’s shortage of GPs and practice nurses will escalate over next decade, report warns. BMJ.

[20] Williamson GR, Rowe L-M, Knowles S, Kane A (2020). Preparation and support for students in community placements: a mixed methods study. Nurse Educ Pract.

[21] Clifford J, Barnes K, Arora R, Raouf S. Leading the Way. The role and value of nurses in general practice in England. In. London. Sonnet Impact; 2021.

[22] Lewis R, Kelly S (2018). GP/GPN partner perspectives on clinical placements for student nurses in general practice: can a community of practice help to change the prevailing culture within general practice?. BMC Fam Pract.

[23] NHS., England: General Practice. Developing confidence, capability and capacity. A ten point action plan for General Practice Nursing. In. London: NHS England; 2017.

[24] Fuller C. Next steps for integrating primary care. In.: NHS England and NHS Improvement; 2022.

[25] NHS England. Standard General Medical Services Contract. In. England: NHS England; 2022.

[26] NHS England. Quality and Outcomes Framework guidance for 2022/2023. In. London: NHS England; 2022.

[27] Calma KRB, McInnes S, Halcomb E, Williams A, Batterham M (2022). Confidence, interest and intentions of final-year nursing students regarding employment in general practice. Collegian.

[28] Calma KRB, Williams A, McInnes S, Halcomb E (2021). New graduate employment in general practice: perceptions of final-year nursing students. Nurse Educ Pract.

[29] Lewis R, Ibbotson R, Kelly S (2019). Student nurses’ career intentions following placements in general practice through the advanced training practices scheme (ATPS): findings from an online survey. BMC Med Educ.

[30] Health Education England. The General Practice Nursing Workforce Development Plan. In. London: Health Education England; 2017.

[31] Tong A, Sainsbury P, Craig J (2007). Consolidated criteria for reporting qualitative research (COREQ): a 32-item checklist for interviews and focus groups. Int J Qual Health Care.

[32] Hackett A, Strickland K (2018). Using the framework approach to analyse qualitative data: a worked example. Nurse Res.

[33] Ritchie J, Lewis J, Nicholls CM, Ormston R (2014). Qualitative research practice: a guide for Social Science Students and Researchers.

[34] Harley B, Cornelissen J (2022). Rigor with or without Templates? The pursuit of Methodological Rigor in qualitative research. Organizational Res Methods.

[35] van de Mortel TF, Needham J, Henderson S (2021). Facilitating learning on clinical placement using near-peer supervision: a mixed methods study. Nurse Educ Today.

[36] Markowski M, Bower H, Essex R, Yearley C (2021). Peer learning and collaborative placement models in health care: a systematic review and qualitative synthesis of the literature. J Clin Nurs.

[37] Lovegrove MJ. Reducing pre-registration attrition and improving retention report. In. London: Allied Health Solutions; 2018.

[38] Dunbar H, Carter B (2017). A sense of belonging: the importance of fostering student nurses’ affective bonds. J Child Health Care.

[39] Patel SE, Chrisman M, Russell CL, Lasiter S, Bennett K, Pahls M (2022). Cross-sectional study of the relationship between experiences of incivility from staff nurses and undergraduate nursing students’ sense of belonging to the nursing profession. Nurse Educ Pract.

[40] Grobecker PA (2016). A sense of belonging and perceived stress among baccalaureate nursing students in clinical placements. Nurse Educ Today.

[41] Suresh P, Matthews A, Coyne I (2013). Stress and stressors in the clinical environment: a comparative study of fourth-year student nurses and newly qualified general nurses in Ireland. J Clin Nurs.

[42] Hamshire C, Barrett N, Langan M, Harris E, Wibberley C (2017). Students’ perceptions of their learning experiences: a repeat regional survey of healthcare students. Nurse Educ Today.

[43] Hamshire C, Willgoss TG, Wibberley C (2013). Should I stay or should I go? A study exploring why healthcare students consider leaving their programme. Nurse Educ Today.

[44] Donnelly C, Ashcroft R, Bobbette N, Mills C, Mofina A, Tran T, Vader K, Williams A, Gill S, Miller J (2021). Interprofessional primary care during COVID-19: a survey of the provider perspective. BMC Fam Pract.

[45] Friman A, Wiegleb Edström D, Edelbring S (2017). Attitudes and perceptions from nursing and medical students towards the other profession in relation to wound care. J Interprof Care.

[46] Bell HT, Granas AG, Enmarker I, Omli R, Steinsbekk A (2017). Nurses’ and pharmacists’ learning experiences from participating in interprofessional medication reviews for elderly in primary health care - a qualitative study. BMC Fam Pract.

[47] Vartanian H, Chou E, Pape Z, DeMuth M, Treat R, Korek S (2022). Twenty questions: increased frequency of social determinants of health addressed with interprofessional student teams in a simulated interview with standardized patients. J Interprofessional Educ Pract.

[48] Brown JB, Reichert SM, Boeckxstaens P, Stewart M, Fortin M (2022). Responding to vulnerable patients with multimorbidity: an interprofessional team approach. BMC Prim Care.

[49] Zhou X-y, Wang Y-f, Dou C-x, Tian X-y, Su J, Chen Y-y, Yan F-x, Yang Q-h, Wang W (2022). Evaluating the effects of simulated interprofessional teaching on the development of clinical core competence in nursing students: a mixed methods study. BMC Nurs.

[50] Mulvale G, Embrett M, Razavi SD (2016). Gearing up’ to improve interprofessional collaboration in primary care: a systematic review and conceptual framework. BMC Fam Pract.

[51] Puffett N, Turley S (2020). How to ensure a successful general practice nursing placement. Prim Health Care.

[52] Dickson CAW, Morris G, Gable C (2015). Enhancing undergraduate community placements: a critical review of current literature. Br J Community Nurs.

